# St. Augustine’s Reflections on Memory and Time and the Current Concept of Subjective Time in Mental Time Travel

**DOI:** 10.3390/bs3020232

**Published:** 2013-04-25

**Authors:** Liliann Manning, Daniel Cassel, Jean-Christophe Cassel

**Affiliations:** 1 Cognitive Neuropsychology and Pathophysiology of Schizophrenia (U1114), Strasbourg University and IFR 37, 12 rue Goethe, 67000 Strasbourg, France; 2 Lycée André Maurois 2 rue du Stade F-67240 Bischwiller, France; E-Mail: cassel.daniel@voila.fr; 3 Laboratory of Cognitive and Adaptive Neuroscience (UMR 7364), Strasbourg University and IFR 37, 12 rue Goethe, 67000 Strasbourg, France; E-Mail: jcassel@unistra.fr

**Keywords:** St. Augustine, subjective time, memory, mental time travel

## Abstract

Reconstructing the past and anticipating the future, *i.e.*, the ability of travelling in mental time, is thought to be at the heart of consciousness and, by the same token, at the center of human cognition. This extraordinary mental activity is possible thanks to the ability of being aware of ‘subjective time’. In the present study, we attempt to trace back the first recorded reflections on the relations between time and memory, to the end of the fourth century’s work, the *Confessions,* by the theologian and philosopher, St. Augustine. We concentrate on Book 11, where he extensively developed a series of articulated and detailed observations on memory and time. On the bases of selected paragraphs, we endeavor to highlight some concepts that may be considered as the product of the first or, at least, very early reflections related to our current notions of subjective time in mental time travel. We also draw a fundamental difference inherent to the frameworks within which the questions were raised. The contribution of St. Augustine on time and memory remains significant, notwithstanding the 16 centuries elapsed since it was made, likely because of the universality of its contents.

## 1. Introduction

Perhaps it might be said rightly that there are three times: a time present of things past; a time present of things present; and a time present of things future. For these three do coexist somehow in the soul, for otherwise I could not see them. The time present of things past is memory; the time present of things present is direct experience; the time present of things future is expectation.

St. Augustine [1], Book 11, Chapter 20, Heading 26.

Klein [2] stated that St. Augustine’s observations in the main belong to the realm of psychological fact. He is one of the rare authors to have acknowledged the influence of St. Augustine’s work on scientific psychology. In the same vein, we attempt in the present study to trace back the psychological facts of a selected notion in current neuropsychology, subjective time in mental time travel, to its medieval origins in the *Confessions*.

We begin by mentioning the importance of subjective time and mental time travel in human cognition and briefly comment on some cognitive-behavioral areas that benefit from research on time-memory relationships. We then suggest the circumstances that might have led St. Augustine to speculate on the concept of the self in past, present and future time. To that end, we briefly comment on some of the ideas that seem to have influenced his work and, most particularly, his method of introspection, and we mention a few examples of fields in psychology that have been influenced by him. We then attempt to draw some comparisons between St. Augustine’s and current descriptions of subjective time through the philosopher’s reflections to convey meaningful answers to the “mystery” of time. Finally, we comment on a matter-of-fact and unbridgeable difference in the theoretical positions from, which questions are asked and interpretations are advanced.

Suggested by Suddendorf and Corballis [3] and Wheeler *et al.* [4], the notion that autobiographical memory operates in the two temporal dimensions has gained ground and become consensual (see [5,6] for reviews). The question about the nature of the time in which mental time travel takes place is central in the characterization of this topic [7]. Different terms have been used to discuss subjective time. Szpunar [7] notes that the notion of chronesthesia [8] emphasizes the awareness of the subjective time in which one’s self exists, while autonoetic consciousness [9] highlights awareness of one’s self existing in subjective time. Time and self are, in every case, inextricably related. Likewise, the notion that remembering the past is achieved to envision the future, rather than to obtain a precise and detailed blueprint of events indicates the harmony of operations of time and self. From a more general standpoint, the reason why we seem to remember the past is considered as the hallmark of flexibility of human cognition and behavior (e.g., [10,11,12]). From an evolutionary standpoint, behavior that increases future survival has a selective advantage, and therefore, mechanisms underlying future thinking are ubiquitous. With regard to those mechanisms, a series of arguments have been posited for or against their presence in humans, exclusively. Corballis [13], for instance, strongly advocates for mental time travel as a case of continuity from animals to humans, with only “a difference of degree and not of kind”. In a different, but closely related perspective, Suddendorf and Corballis [3,14] and Suddendorf and Busby [15] point out the prerequisite capacities for mental time travel, such as the ability to represent our representations as representations. This ability allows the appropriate use of open-ended generativity that is central in the combination and recombination of a finite set of elements (see below, the “constructive episodic simulation hypothesis”). It has been suggested that at some point in the course of phylogenesis, it was the social pressure that drove the evolution of intelligence. Pinker (in Suddendorf and Coballis, [14]) stated that much of what humans recall and foresee has to do with “who did what to whom, when, where and why”. In this same perspective, some studies of personality also underscore the importance of the social, everyday relationship to one another. Uher [16], for example, stated that people encode in their everyday languages all the differences in other people that they perceive as most salient in ordinary encounters and that they consider to be socially relevant.

The adaptive value to cognition and behavior of mental time travel has been actively investigated the last decade. Results are expanding the way in which basic and applied researchers characterize a wide variety of scientific issues. Thus, Atance and O’Neil [17] argued that time-memory relations, particularly, episodic future thinking represent an important organizing construct in cognition, since it can hold clarifying value in topics, such as prospective memory, goal pursuit and the planning fallacy. Likewise, Szpunar [12] comments on cognitive behavioral therapy reports, which indicate reduction of problem behaviors after appropriate future simulation. In a different perspective, Klein *et al.* [18] suggest that an evolved organism behave more adaptively at a later time because of experiences at an earlier time, owing to cognitive mechanisms that use earlier information to plan behavior (see above for some comments on evolution and prerequisite capacities for mental time travel). To mention one more area of research, mental time travel has been deemed to be necessary for normal development and independent living [19]. To find out further cognitive capacities that either depend on or are influenced by the ability of projecting oneself in different temporal dimensions is likely one of the most exciting challenges in current cognitive neuropsychology.

With regard to the history of the concept, the vast majority of reviews on mental time travel begin with Tulving’s episodic/semantic distinction in 1972 [20], and only a few mention the nineteenth century (e.g., Klein [11] cites an astonishingly contemporary statement by Bradley, who, in 1887, said that our memory is directed towards “the side from which change comes” because of the practical necessity to “go the way of anticipation”). In the rest of the paper, we refer to some of St. Augustine’s introspective reflections that could be considered as the first inklings of the notion of subjective time in mental time travel.

## 2. The Context of St. Augustine Reflections

The first mention of the power of transcending the irreversibility of (physical) time thanks to memory is in Hesiod’s Theogony; in the eighth century BC. St. Augustine was probably familiarized with Greek mythology and Greek philosophy, but contrary to what we suggested in our previous work [21], he may have not been acquainted with Hesiod’s oral tradition or, at least, the Theogony was not in his conscious mind when he struggled all along the 31 chapters of Book 11 to grasp the meaning of time (in chapter 22, after deep questioning, he says: “*My soul burns ardently to understand this most intricate enigma*”). More importantly, Plotinus’ way of reflecting seems to have greatly influenced St. Augustine’s conceptual elaboration on memory and time [22]. Both Plotinus and St. Augustine can be thought of as early introspectionists, because of their interest in the human being’s inner life. Subjective observation, introspection or awareness of one’s own mental processes was named “the interior man’s sense” by opposition to the traditional five senses meant to process the outer world. The human being, in the Augustinian conception, is considered from the inner sense perspective and possesses, consequently, self-consciousness (see below).

Being familiarized with Greek philosophy was probably decisive in St. Augustine’s reasoning to bypass difficulty in trying to apprehend the essence of the past (pastness). He overcame the impasse, initiating a new perspective in his reflections that resulted in memory of the past bearing relation to anticipation of the future and presence of the present. This *volte-face* in his way of conceptualizing memory determined both the development of his notion of time in Book 11 of the *Confessions* [23] and the unique place he gave to memory in Book 10 [24]. Indeed, owing to the ephemeral and fleeting nature of the present, memory becomes in St. Augustine’s method of introspection, the pivotal entity through which one can think meaningfully of temporal continuity. Experienced continuity is possible only by and through memory.

On his part, St. Augustine has influenced the study of fields as different as the relations between bodily processes and sensation or the domain that is of particular importance in the present study, the introspective principle. Indeed, the description of St. Augustine’s introspection became, in Kantor’s words [25], the establishment of many of the basic doctrines of psychology. They started as doctrines of the soul to become doctrines of the mind, consciousness and thought. They are all characterized by the primacy of the person, *i.e.*, the assumption that there is something unique about the human organism: a principle or a power that differentiates it from the rest of the world, *i.e.*, self-consciousness. A further central domain influenced by St. Augustine’s reflections and one that is inextricably related to subjective time is the sense of self: the absolute uniqueness of the mind, developed below.

## 3. St. Augustine’s Introspective Descriptions on Time and Memory

St. Augustine, as he stated throughout his whole work, endeavored to know God, and consequently, introspection on memory and time was scrutinized by means of questions, comments and requests addressed to that end (see [21]). From this perspective, St. Augustine developed the observation of his own learning, recollection and experience of time. The result of this purely introspective venture, still respected 16 centuries later, can be useful, because of the depth of analysis and the accuracy of descriptions. Guided by current notions of subjective time in mental time travel, we have selected a few meditations (a minute fraction of his monumental work).

In Book 11 [1], St. Augustine ascertains the difference between the Creator’s eternity and the creature’s time. Beginning with the latter, in chapter 6, heading 8 (for all quotation from Book 11, only the section corresponding to the chapter, the first figure, and the heading, the second figure, appear in the remaining of the text; the sole quotation from Book 10 indicates also the Book): “*The syllables sounded and passed away, the second after the first, the third after the second, and thence in order, till the very last after all the rest; and silence after the last. From this it is clear and plain that it was the action of a creature, itself in time, which sounded that voice…*”. With regard to God’s time, St. Augustine adds (in section 7.9): “*For what was first spoken was not finished and something else spoken until the whole series was spoken; but all things at the same time and forever*. Finally (in section 14.17), St. Augustine summarizes these differences in a brief outstanding description: “*If the present, should always be present, and never pass into time past truly it should not be time, but eternity*”. Being one of the fathers of the church, it was imperative for St. Augustine to let his readers know that his quest and questions were addressed to understand human time. By the same token, he resolved the difficult question of the present. While for St. Augustine it is tantamount of eternity, for James [26], the present is an ideal abstraction not only never realized in sense, but probably never even conceived of by those unaccustomed to philosophical meditations. On his part, McTaggart’s [27] conceived time in two distinct ways called A- and B-series. The former establishes that every moment is either past or present or future, while the latter determines that every moment is earlier or later than each other moment. A parallel between A- and B-series and human time and eternity in the *Confessions* is tantalizing. Indeed, with regard to B-series, since “M is ever earlier than N”, it involves no change and, therefore, cannot be an account of time: facts about B-series are eternal. Facts about A-series, on the contrary, are always changing from future to present and from present to past. They are then a proper account of time, said McTaggart. Opposing unchanged present, *i.e.*, eternity in B-series to constant change in A-series seems to be an echo from the Augustinian conclusion (in section 7.9):“*For otherwise, we should have time and change and not a true eternity*”. McTaggart, later on, described a tautology inherent in the A-series; he could not escape to the contradictions in the physical relation of past, present and future. St. Augustine had resolved this impasse by ascribing the changes in time to “*the action of a creature, itself in time”* (see above).

The first steps towards reasoning in terms of subjective time might have originated in a different domain: St. Augustine’s meditation on the impossibility of eliminating doubt. These meditations have been considered to be one of the finest illustrations of his inner thought descriptions. In that, he not only anticipated the famous Cartesian conclusion, but as Klein [2] stated, he may even have been more accurate than Descartes, since St. Augustine’s conclusion was: I doubt, therefore I am. With the process of doubting as a self-conscious event, St. Augustine introduced the separation between subjective and objective observation in psychology. This is essential in several approaches and, particularly, in the study of time perception either as a psychophysical, chronological and unidirectional process or, on the contrary, as the self-conscious, memory-based, subjective phenomenon, *i.e.*, subjective time.

However, what is subjective time in twenty-first century? To respond to this complex question, Szpunar [7] begins by saying what subjective time is not. It is not the time studied by physical sciences, because “past” and “future” do not exist in the physical reality. Subjective time is, therefore, necessarily defined in relation to a sentient observer. Time past and time future are experienced with a sense of a unitary self since past and future exist only in the human mind.

How do the *Confessions* tackle this difference in types of times? An early observation in Book 11 about the temporal dimensions, past, present and future is close to a physical description (in section 11.13): “*Therefore, let it see that all time past is forced to move on by the incoming future*”. In 1890, James [26] started his own reflections on time by stating that the lingerings of the past drop successively away and the incomings of the future make up the loss. More to the point (in section 16.21), St. Augustine acknowledges the two different types of time: “*We measure the passage of time when we measure the intervals of perception. But who can measure times past which now are no longer or times future which are not yet?*” This distinction guides his reflections towards the mind (in section 18.23): “*For if there are times past and future, I wish to know where they are… Wherever they are and whatever they are they exist only as present. Although we tell of past things as true, they are drawn out of the memory, not the things themselves, which have already passed, but words constructed from the images of the perceptions which were formed in the mind… My childhood, for instance, which is no longer, still exists in time past which does not now exist. But then I call to mind its image…”* St. Augustine follows his meditation by tackling the future (in section 18.24): “*Whatever may be the manner of this secret foreseeing of future things, nothing can be seen except what exists* (…) *I see the dawn; I predict that the sun is about to rise. What I see is in time present, what I predict is in time future because it is not yet. Yet I could not predict even its rising unless I had an image of it in my mind*”. St. Augustine’s quest on time was defined, to some extent, by measure. He establishes that before measuring the thing, it must be present to be seen: “*Future events, therefore, are not yet. And if they are not yet, they do not exist. And if they do not exist, they cannot be seen at all, but they can be predicted from things present, which now are and are seen*” (also in section 18.24). Interestingly, in a different part of the Book (section 27.36), his example, a series of sounds, is not physically encountered, but imagined. From this image, anticipation takes place and, more important, anticipation is committed to memory: “*If anyone wishes to utter a prolonged sound and if, in forethought, he has decided how long it should be, that man has already in silence gone through a span of time and committed his sound to memory*”. Two different aspects of reflection in relation to subsequent studies of mental imagery are present in this quotation, (i) memories of imagined events and (ii) how to measure mental images. With regard to the first point, Ingvar [28] coined the phrase “memory of the future”. More recently, based on associative processing, whose primary outcome is the generation of predictions, Bar [29] pointed out that the central role of mental time travel is to create scenarios by combining past memories and future projections and to store those “mental memories” that will be used similarly to real memories, to provide scripts for plausible future situations. Moreover, on the bases of the acknowledged role of the hippocampus in associative encoding, Martin *et al.* [30] interrogated the ability to generate imagined episodic details and that of encoding and remembering the imagined events. Indeed, potential benefits of imagining the future derive from the ability to retrieve the “mental memories” and refer to in future behavior. Martin *et al.* suggested that imagining the future entails a greater degree of encoding relative to previously stored information, owing to the generation of new mental representations. A further step in the understanding of memory of the future comprises the study of the impact of the emotional valence on memory of imagined future events. Szpunar *et al.* [31], investigating this characteristic of mental time travel, found a positivity bias when people remember imagined positive, negative and neutral events. We turn presently to the second aspect of the quotation. In this paragraph, St. Augustine describes how to measure mental images and by so doing, confers reality to them. *“…we measure periods of time as they pass. And if anyone asks me, ‘How do you know this?’ I can answer: ‘I know because we measure”.* Several famous psychologists followed a similar reasoning, but different method. Thus, James [26] reports that like himself, Wundt and one of his students had tried to measure the present. This was defined as the maximum extent of our immediate distinct consciousness for successive impressions. James also relates Exner’s measures of the present in different sensory modalities. Notwithstanding the efforts to determine experimentally subjective time, the conclusion is similar to St. Augustine’s, that is, that to be conscious of a time interval at all is one thing, to tell whether it be shorter or longer than another interval is a different thing. However, what if the philosopher must remain within the realm of meditations with no experiments whatever? St. Augustine relates measure to temporal dimensions as follows (section 21.27): “*We could not measure things that do not exist, and things past and future do not exist (…). Therefore, from what is not yet (future) through what has no length (present), it passes into what is no longer (past). But what do we measure, unless it is a time of some length? For we cannot speak of single, and double, and triple, and equal, and all the other ways we speak of time, except of the lengths of the periods of time. But in what ‘length’ then do we measure passing time?*” Faced with these impasses, St. Augustine’s interim conclusion is as follows (section 26.33): “*From this it appears to me that time is nothing other than extendedness; but extendedness of what I do not know. This is a marvel to me”.* In addition, his conclusion: *“The extendedness may be of the mind itself*”. This conclusion has influenced philosophers and psychologists across centuries. As an example, it is found in Locke’s Essay [32], where he explains that as far as the consciousness can be extended backwards to any past action or forwards to actions to come, so far reaches the identity of the person. In this new perspective, to measure is not only unnecessary, but impossible, as the items to be measured have no longer an established order of succession. James [26] gives an example: If idea A follows idea B, consciousness simply exchanges one with another. That B comes after A is for our consciousness a non-existent fact. The echoes of the extendedness of the mind, present in the nineteenth century, are also present in current neuropsychology [7,11,12,33]. Dalla Barba [33], for instance, emphatically criticizes what he calls the paradox of the memory trace. His central argument is that objects and events acquire a temporal dimension, past or future, only in the presence of a person. Consciousness of past things (or future things) is neither contained in a physiological nor cognitive trace, but experienced in a phenomenological way. Nyberg *et al.* [34] carried out an investigation to tell apart the phenomenal experience and the conscious temporality of the experience. They showed that imagining oneself carrying out a familiar activity at the present time, imaging the same task done yesterday or tomorrow activated the left lateral parietal cortex, the left frontal cortex, the right cerebellum and the thalamus. The interest of Nyberg *et al.*’s design derives from the fact that holding constant the phenomenal experience (a familiar activity), they obtained a pattern of cerebral activations different from that observed in investigations in which phenomenal experience is at study and in which a relatively consensual observation is a hippocampal involvement. The rich and diverse current investigations, a few examples of which are commented in relation to St. Augustine’s notions of time, have been considered by Schacter *et al.* [35] in an extensive review on memory and subjective time, in which the authors show the way of future research. For Tulving and Szpunar [36], the most important aim ever in neuroscience’s future research is clearly stated. The authors tackle a different facet of subjective time, the “apparent paradox” that it engenders. Indeed, something that does not exist in physical reality plays a very important role in governing and regulating something that does exist, *i.e.*, human affairs. To try and resolve the paradox, they propose the existence of two realities, mental (the mind) and physical (the brain). The relations between them are complex, since the mind depends on the brain, but the mind also transcends the brain. The intricate question of searching the common “thing” the mind and the brain are “made of” seems to be inescapable. In section 20.24, St. Augustine makes it clear that the future events are imaginable exclusively because they are conceived in the mind: “*When, therefore, they say that future events are seen, it is not the events themselves (…), but perhaps, instead, their causes and their signs are seen, which already do exist. Therefore, to those already beholding these causes and signs, they are not future, but present, and from them future things are predicted because they are conceived in the mind*”. In the same vein, St. Augustine’s reflections on awareness and attention (being conscious of), describes one of the finest examples of the phenomenological description in section 28.38: “*I am about to repeat a psalm that I know. Before I begin, my attention is extended to the whole; but when I have begun, as much of it as becomes past by my saying it is extended in my memory; and the life of this action of mine is divided between my memory, on account of what I have repeated, and my expectation, on account of what I am about to repeat; yet my consideration is present with me, through which that which was future may be carried over so that it may become past. Which the more it is done and repeated, by so much (expectation being shortened) the memory is enlarged, until the whole expectation be exhausted, when that whole action being ended shall have passed into memory*”*.*

Intriguingly, in Book 10 (section 8.14), St. Augustine had already illustrated the extendedness of the mind in the following, far reaching meditation: “*Out of the same storehouse, with these past impressions, I can construct now this, now that, image of things that I either have experienced or have believed on the basis of experience—and from these I can further construct future actions, events and hopes; and I can meditate on all these things as if they were present…”*

St. Augustine was very likely the first philosopher to put forward the idea that past and future could be seen as equivalent entities that exist, as long as they are present in our consciousness. From the 1980’s with Tulving works, the notion of a common neuro-cognitive platform for past and future was proposed on a theoretical basis [3,9] and, later on, a series of results obtained using functional neuroimaging [37,38,39,40]. Within this context, Schacter and Addis [41] put forward the “constructive episodic simulation hypothesis”, which states that a memory system that allows using stored information in a flexible manner for imagination of future events, as St. Augustine’s elegant metaphor describes, must be essentially a constructive memory system. The first inklings of the dynamic nature of autobiographical memory system are found in Bartlett’s works [42]. He stated that remembering, particularly in a social context, serves to share our impressions with others, so that people embellish upon their recollections. This notion was later on developed by Conway [43], who suggested the principle of “coherence” between one’s life experiences and the representation of oneself. Conway argued that over time, coherence takes precedence over the principle of “correspondence”, which refers to conformity of memories of one’s experiences with reality.

## 4. Frameworks of Interpretation

Current authors working on mental time travel recognize in this notion, an adaptive implication for behavior. The adaptive dimension is the fact of potentially being prepared for near future situations on the bases of past experience. Thus, Klein *et al.* [44] suggested that the adaptive function of information storage is intrinsically prospective. Interestingly, Klein *et al.* proposed that mental time travel should not be limited to episodic memory, since the two components of retrograde memory, episodic and semantic are interactive and they are not reducible either empirically or conceptually. It follows, naturally, two distinct times, “lived time” made of episodic past—future events and “known time” made of semantic mental time travel. On their part, Buckner and Carroll [10] put forward a strong case for distinct functions that both used past experiences for mental exploration of the future (among diverse departures from the present) and relied on a common set of processes they called “self-projection”. The authors’ starting point was the existence of a shared brain network to support different forms of the concept of self-projection (but, see [7]). In every case, a central processing component is mental simulation based on personal past experiences that allows the exploration of alternative perspectives. One “leaves”, so to speak, time present or a given place or one’s own personal perspective to explore alternatives, through mental time travel, spatial navigation and the theory of mind, respectively. All alternatives have in common a mental preparation for what might lie ahead. Future planning requires a flexible cognitive system that is assumed to be adaptive (preparing for the future is a vital task in any domain important for survival). The “Self-projection” proposal suggests that mental time travel is only part of a more complex process and emphasizes that thinking ahead is the vital process. Moreover, Bar [29,45] contributed to the same notion, on the bases of analogical thinking. He suggested that when we see a new object or we meet a person for the first time or encounter a new social situation, we follow a recognition-by-analogy process. We use experience (“it looks like...”; “he reminds of...”) and the associations that accompanied those initial representations in this mental process. Predictions based on analogy (“that must be a …”; “he must be nice/ rigid/ unpleasant as...”) might be seen, therefore, as restraining the selection of what aspects of the environment will be privileged and how they will be interpreted. Analogies imply associations: Bar [29,45,46] proposed associative processing as the fundamental operation of our mental life. Associations, the units of thoughts, are used to generate predictions in an uninterrupted flow. More recently, Szpunar [12], in his comprehensive review on future thought, calls our attention, among other points, to the fact that it represents a frequently occurring mental phenomenon that has clear adaptive implications for behavior. Complementarily, anticipation in the form of predictions determines which past recollections are ‘alive’.

St. Augustine’s introspective reasoning on time past and time future, being rooted as it was in the context of his quest of God, could not and did not include any purely human considerations. Although his introspection led him to express essential points of the subject, there could not be any hint of any potential usefulness of the capacity of travelling in subjective time. The twentieth century philosopher, Sartre [47], considered that the essential in man (*le propre de l’homme*) was not to know God, but anticipation. Being an atheist, he bypassed St. Augustine’s ontological questions. Sartre integrated the theory of anticipation to his phenomenological standpoint on the bases of the notion of intentionality [48] He went from the Augustinian “I doubt, therefore I am” (and the Cartesian “I think, therefore I am”) to his own conclusion: “I think, therefore I anticipate”. In Sartre’s notion of time, our memory “uses” the three temporal dimensions, with no separation for we have “these pasts, these presents and these possible futures, all at once”. Past, present and future interpenetrate the mind, and therefore, we are continuously reorganizing the past, present and future, that is, forgetting, restoring and imagining events. Sartre focused on the human mind, from the perspective of existentialism and was able to give a crucial role to mental time travel: “It is the future that decides if the past is alive or dead. (…). Thus, the order of my future choices will determine the order of my past, and this order is by no means chronological” [47].

## 5. Concluding Comments

In summary, about 16 centuries elapsed between St. Augustine’s meditations and the proposal of a ‘mental time travel’ concept. Impervious to chronological time, it appears that St. Augustine’s *Confessions* still are and very likely will remain a valuable source of reflection. Two of the reasons may be his outstanding capacity of introspective analysis and the universality of its contents, *i.e.*, memory and subjective time. St. Augustine’s meditations, particularly on the continuity of the self, seem close to ours and their influence on our way of reflecting on mental time travel must have been immense (but never acknowledged). His considerations of time future could not include the adaptive quality that is the hallmark of current analysis on future thinking. However, in his own perspective, St. Augustine alludes to a beneficial quality of time future (in section 19.25), realizing that God taught him things present from the signs of things future.
